# Draft genome sequence of *Yarrowia lipolytica* NRRL Y-64008, an oleaginous yeast capable of growing on lignocellulosic hydrolysates

**DOI:** 10.1128/MRA.00435-23

**Published:** 2023-11-20

**Authors:** Sujit Sadashiv Jagtap, Jing-Jing Liu, Hanna E. Walukiewicz, Robert Riley, Steven Ahrendt, Maxim Koriabine, Kelly Cobaugh, Asaf Salamov, Yuko Yoshinaga, Vivian Ng, Chris Daum, Igor V. Grigoriev, Patricia J. Slininger, Bruce S. Dien, Yong-Su Jin, Christopher V. Rao

**Affiliations:** 1 Department of Chemical and Biomolecular Engineering, University of Illinois at Urbana–Champaign, Urbana, Illinois, USA; 2 DOE Center for Advanced Bioenergy and Bioproducts Innovation, University of Illinois at Urbana–Champaign, Urbana, Illinois, USA; 3 Lawrence Berkeley National Laboratory, US Department of Energy Joint Genome Institute, Berkeley, California, USA; 4 Department of Plant and Microbial Biology, University of California, Berkeley, California, USA; 5 Bioenergy Research Unit, National Center for Agricultural Utilization Research, USDA-ARS, Peoria, Illinois, USA; 6 Department of Food Science and Nutrition, University of Illinois at Urbana–Champaign, Urbana, Illinois, USA; University of California, Riverside, California, USA

**Keywords:** *Yarrowia lipolytica*, genome analysis, oleaginous yeast

## Abstract

*Yarrowia lipolytica* is an oleaginous yeast that produces high titers of fatty acid-derived biofuels and biochemicals. It can grow on hydrophobic carbon sources and lignocellulosic hydrolysates. The genome sequence of *Y. lipolytica* NRRL Y-64008 is reported to aid in its development as a biotechnological chassis for producing biofuels and bioproducts.

## ANNOUNCEMENT

*Yarrowia lipolytica* NRRL Y-64008 is an oleaginous yeast that is generally recognized as a safe organism (1, 2). It is a promising host for high-titer production of sugar alcohols, citric acid, lipases, proteases, and lipid-based chemicals (3–6). It grows in the presence of organic acids at low pH and ionic liquids and is salt-tolerant (7–9). *Y. lipolytica* can grow on sugars and hydrophobic substrates and produce fatty acid-based bioproducts (10, 11). It can also produce lipids from lignocellulosic hydrolysates (12–14). It was originally isolated from the soil (7). This yeast was named *Y. lipolytica* NRRL Y-64008 after a BLAST analysis of its internal transcribed spacer (ITS) sequence (GenBank accession number ON114154.1) against the UNITE database (https://unite.ut.ee/) and genome sequencing results. Interesting phenotypes observed for this strain include the ability to utilize plant-based sugars and growth on undetoxified biomass hydrolysates (12–14). We sequenced the genome of *Y. lipolytica* NRRL Y-64008 to allow for further research into its metabolism and enable metabolic engineering to produce biofuels and bioproducts from renewable carbon sources.

The cells were grown for 2 days at 30°C and 250 rpm in a YPG medium (10 g/L yeast extract, 20 g/L peptone, and 20 g/L glucose) and harvested at an OD_600_ of 10. Genomic DNA was extracted using the Dr. GenTLE (from yeast) High Recovery Kit (Takara Bio Inc., Shiga, Japan).

Libraries larger than 10 kb for PacBio sequencing were prepared using 5 µg of genomic DNA (15, 16). The sheared DNA was treated with exonuclease, followed by end repair and blunt adapter ligation with the SMRTbell Template Prep Kit 1.0. BluePippin (Sage Science) was used to select the library at 6 kb cutoff, which was purified with AMPure PB beads. Following the annealing of the PacBio sequencing primers to the SMRTbell template library, a Sequel Binding Kit 2.0 was used to bind the sequencing polymerase to the primers. On a Sequel II sequencer from Pacific Biosystems, the SMRTbell template libraries were then sequenced using a v3 sequencing primer, 8M v1 SMRT cells, and version 2.0 sequencing chemistry with 1 × 900 sequencing movie run durations. Reads containing inverted repeats were removed with BBTools version 38.79 (https://jgi.doe.gov/data-and-tools/bbtools/), and the default minimum read length was 40 bp. Filtered circular consensus sequencing(CCS) reads were assembled with Flye version 2.3.6 release (https://github.com/fenderglass/Flye) (-g 40M --asm-coverage 50 --pacbio-corr) and polished with arrow version SMRTLink v8.0.0.80529 (https://www.pacb.com/support/software-downloads). All CCS reads were used in both assembly and polishing. Assembled scaffolds were screened using BLAST vs the National Center for Biotechnology Information (NCBI) RefSeq and nt databases to ensure that no non-fungal contaminants were assembled.

The 20.5 Mb genome assembly was in six contigs (*N*_50_ value of 3 Mb), with a 149.31× sequencing read coverage depth and a 49.04% GC content. A combination of gene predictors including transcriptome-based, protein homology-based, and GeneWise seeded by BLASTx alignments of fungal sequences in the NCBI’s non-redundant protein database, and *de novo* and GeneMark was applied to a repeat-masked genome assembly (17–21). Functional annotations were inferred from the best BLAST hits against the NCBI non-redundant protein databank and InterProScan (22). The JGI Annotation process was used to predict 6,577 protein-coding genes from the genome (23).

## Data Availability

MycoCosm (https://mycocosm.jgi.doe.gov/YarliY64008_1) provides access to the whole-genome assemblies and annotation. This Whole Genome Shotgun project has been deposited at DDBJ/ENA/GenBank under the accession number JAKETR000000000. The version JAKETR000000000.1 is discussed in this paper. The accession numbers for the project and reads are PRJNA791961 and SRR17752527, respectively.
